# Revision of the genera *Hovadelium* Ardoin and *Mimolaena* Ardoin (Coleoptera, Tenebrionidae, Laenini) from Madagascar, with remarks on tribal assignment^1^

**DOI:** 10.3897/zookeys.326.5871

**Published:** 2013-08-26

**Authors:** Wolfgang Schawaller

**Affiliations:** 1 Staatliches Museum für Naturkunde, Rosenstein 1, D-70191 Stuttgart, Germany

**Keywords:** Coleoptera, Tenebrionidae, Lagriinae, Laenini, *Hovadelium*, *Mimolaena*, taxonomy, new species, Madagascar

## Abstract

The genera *Hovadelium* Ardoin, 1961 and *Mimolaena* Ardoin, 1961, endemic in Madagascar, are revised and assigned to the tribe Laenini Seidlitz, 1896 (subfamily Lagriinae Latreille, 1825). New species: *Hovadelium ardoini* **sp. n.**, *Hovadelium bremeri* **sp. n.** and *Mimolaena janaki* **sp. n.** An identification key is compiled for all taxa. Distribution of *Hovadelium* (5 species) and *Mimolaena* (3 species) is mapped. The congeners might be indicator species for the highly endangered mature forests in Madagascar.

## Introduction

Ardoin (1961) described the genera *Hovadelium* Ardoin, 1961 (type species *Hovadelium discoidale* Ardoin, 1961) and *Mimolaena* Ardoin, 1961 (type species *Mimolaena pauliani* Ardoin, 1961), endemic to Madagascar, and placed them into the tenebrionid tribe Adeliini. Subsequently, additional species were described by Ardoin (1976) and Ferrer (1998). In the revision of the tribe Adeliini, Matthews (1998) mentioned, that the tribal assignment of the Malagasy genera *Hovadelium* and *Mimolaena* either to Adeliini or to Laenini remains doubtful. Both genera are placed now herein finally into Laenini because of the lack of defensive glands. Different genera of this tribe are also known from South Africa (Endrödy-Younga and Schawaller 2002, Ferrer 2005).

So far, all descriptions were based only on single specimens. Recently, a huge number of newly collected specimens, mainly of *Hovadelium*, were handed over to the author for examination by Prof. Dr. H. J. Bremer (Osnabrück, Germany). This material, including three so far undescribed species, is represented herein, together with reexamination of the previously described taxa.

**Figure 1.**
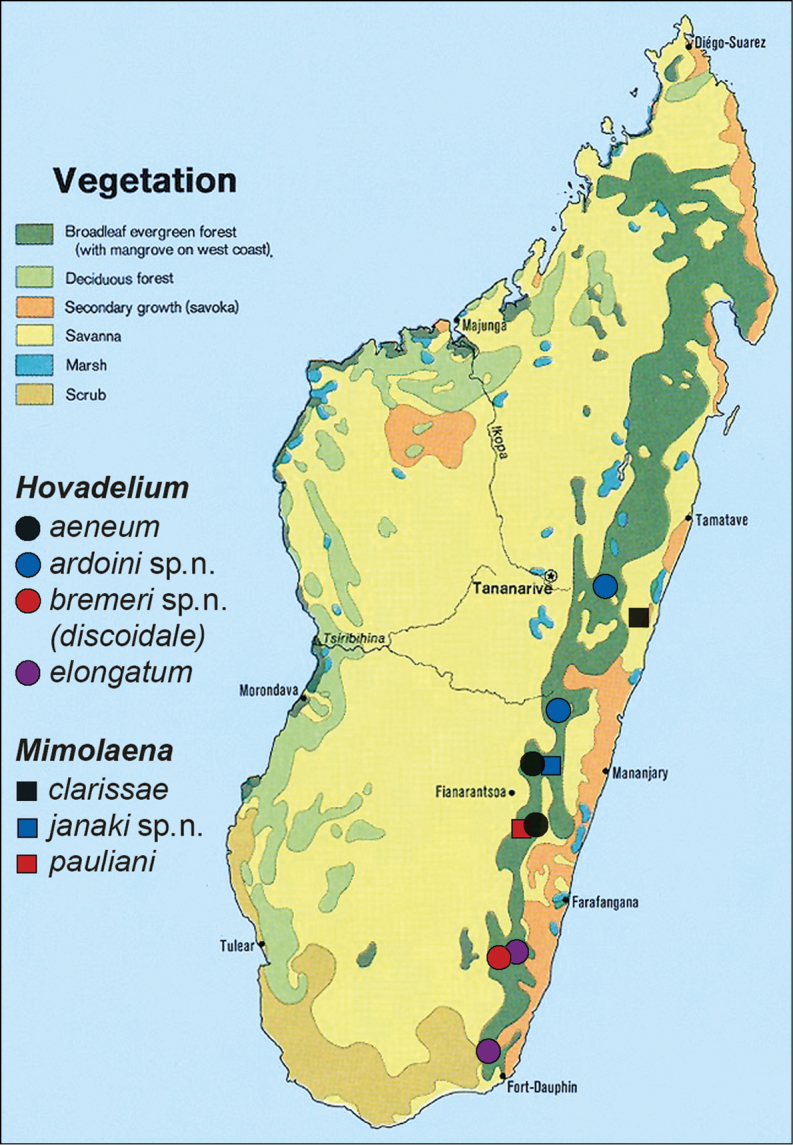
Records of Laenini in Madagascar, vegetation map (modified from NationMaster.com). *Hovadelium discoidale* could not be mapped (known only from “Madagascar” without detailed locality).

As other members of Laenini, all species are wingless and have restricted distributional patterns (Map see Fig. 1). So far, all records originate from the southeastern part of the island, additional taxa might be present in the northeastern part. Living in litter of the broadleaved evergreen forests, the congeners can be considered as indicator species for these mature and highly endangered forests in Madagascar.

## Depositories

CRFL Collection René Fouquè, Liberec, Czech Republic

HNHM Hungarian Natural History Museum, Budapest, Hungary

MNHN Muséum national d’Histoire naturelle, Paris, France

MZUF Museo Zoologico de “La Specola”, Firenze, Italy

NMPC National Museum, Prague, Czech Republic

SMNS Staatliches Museum für Naturkunde, Stuttgart, Germany

TMSA Ditsong National Museum of Natural History, Pretoria, South Africa

ZSM Zoologische Staatssammlung, München, Germany

## Tribal assignment

Some specimens of *Hovadelium ardoini* sp. n. were sent to E. Matthews (Adelaide) for a personal dissection of the female genital tract. The examination showed, that defensive glands are completely absent in all the dissected specimens, which is characteristic for Laenini and the only difference to Adeliini.

Matthews (in litteris): “I have dissected the females and can’t see any trace of defensive glands, certainly not the long ones between segments 8 and 9 which are found in all Adeliini. There are no 7/8 glands either, although finding those in Laenini would not be surprising since they are characteristic of many Lagriinae. Stridulatory files (plectron) are absent. There are also no vaginal sclerites, such as the ones I found in one *Laena* (and most Adeliini). The spermatheca consists of three short wide tubules on the side of the vagina, similar to those of a species of *Laena* (Matthews 1998: fig. 57) which however has two long narrow tubules. Too few Laenini have been dissected for us to know the significance of these details, but the general configuration of the female system is typical of Laenini/Adeliini. The aedeagus of the male is of the usual simple type.”

Ovipositor and female genital tract (Fig. 15): Paraproct and coxite subequal in length, coxite lobes 3 and 4 fused, digitiform, gonostyles terminal in position, coxite baculi transverse, paraproct baculi longitudinal, spiculum gastrale a slender rod without terminal fork. No bursa copulatrix, three short and wide spermathecal tubules attached to side of vagina, vaginal sclerites absent, long slender spermathecal accessory gland attached to anterior end of vagina.

### The genus *Hovadelium*

#### Hovadelium aeneum

Ardoin, 1961

http://species-id.net/wiki/Hovadelium_aeneum

Figs 2 , 10

##### Reexamined type-material.

C Madagascar, Plateau Soaindrana, Andringitra-Ambalavao, 2090 m, 16.I.1958, leg. R. Paulian, ♀ holotype MNHN.

##### New material.

C Madagascar, Andringitra, Andohariana, 2000–2100 m, mission C.N.R.S., 1 ex. MNHN (det. Ardoin). – E Madagascar, Massiv Ambondrombe, Ikoka, 1300–1400 m, 12.–13.III.1996, leg. J. Janák & P. Moravec, 11 ex. SMNS, 5 ex. ZSM. – E Madagascar, Massiv Ambondrombe, 1300–1400 m, 14.III.1996, leg. J. Janák & P. Moravec, 11 ex. SMNS, 5 ex. TMSA, 5 ex. ZSM. – E Madagascar, Massiv Ambondrombe, cote 1579, 1500–1600 m, 15.–18.III.1996, leg. J. Janák & P. Moravec, 11 ex. SMNS, 5 ex. HNHM, 5 ex. NMPC, 5 ex. ZSM. – E Madagascar, Massiv Ambondrombe, 1700 m, 17.III.1996, leg. J. Janák & P. Moravec, 1 ex. SMNS.

##### Diagnostic characters.

Body length 2.7–4.0 mm (the holotype has a length of 4.0 mm and not of 5.0 mm as given by Ardoin 1961). Pronotum subquadrate, widest before the middle, with rounded anterior and posterior angles, surface slightly convex, with fine and sparse punctation, between punctation slightly shagreened. Elytra with punctural rows in striae, intervals convex, slightly shagreened and with an irregular row of tubercles, interval 7 at base near shoulders with a longer seta. Aedeagus see Fig. 10.

**Figures 2–5.**
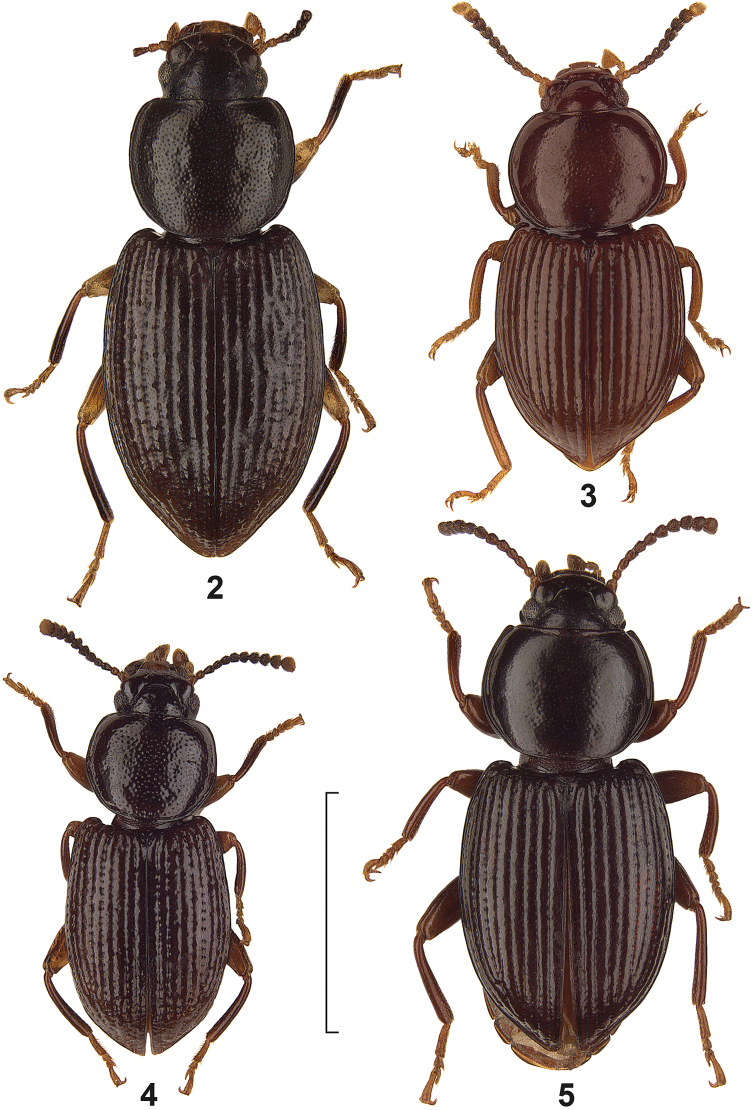
Dorsal view of *Hovadelium* species. **2** *Hovadelium aeneum*, holotype MNHM **3** *Hovadelium ardoini* sp. n., paratype SMNS **4** *Hovadelium bremeri* sp. n., holotype SMNS **5** *Hovadelium discoidale*, holotype MNHN. – Scale line 2 mm.

#### Hovadelium ardoini sp. n.

http://zoobank.org/9E7A178D-AC88-4A2A-A947-5A05D8931C50

http://species-id.net/wiki/Hovadelium_ardoini

Figs 3 , 11

##### Type specimens.

Holotype male: E Madagascar, Fianaratsoa Prov., Ambohimahamasoa, 1300–1400 m, 21.–23.III.1996, leg. J. Janák & P. Moravec, SMNS. – Paratypes: Same data as holotype, 20 ex. SMNS, 5 ex. HNHM, 5 ex. TMSA, 5 ex. ZSM. – E Madagascar, Ranomafana NP, Sahavondrona, 1150–1250 m, 3.–4.II.1995, leg. J. Janák, 13 ex. SMNS, 5 ex. NMPC, 5 ex. ZSM. – E Madagascar, Ranomafana NP, Vohiparara, 1100–1200 m, 21.–24.I.1993, leg. J. Janák, 2 ex. SMNS. – E Madagascar, Fianaratsoa Prov., Ranomafana, 29.XI.–2.XII.1995, leg. I. Jeniš, 1 ex. SMNS. – E Madagascar, Maromiza, Andasibe (Périnet), 1000–1200 m, 9.II.1993, leg. J. Janák, 1 ex. SMNS. – E Madagascar, Maromiza, Andasibe (Périnet), 930–1000 m, 7.–10.I.1995, leg. J. Janák, 5 ex. SMNS. – E Madagascar, Maromiza, Andasibe (Périnet), 7.XI.1998, leg. R. Müller, 1 ex. TMSA.

##### Diagnosis.

*Hovadelium ardoini* sp. n. is similar to *Hovadelium discoidale* Ardoin, 1961, but lacks the striking deep groove ventral of the eyes. Both can be separated also by the shape of the pronotum widest behind the middle in *Hovadelium discoidale* (Fig. 5), but widest in the middle in *Hovadelium ardoini* sp. n. (Fig. 3), and by the anterior angles of the pronotum, which are distinctly marked in *Hovadelium discoidale*, and completely rounded in *Hovadelium ardoini* sp. n. The elytral punctural rows are identical in both species, but the disc of the elytra is flattened in *Hovadelium discoidale*, whereas in *Hovadelium ardoini* sp. n. the elytra are more convex. See also under *Hovadelium bremeri* sp. n. and key below.

##### Description.

Body length 3.3–4.7 mm, unicoloured dark brown. Eyes flat, not prominent; without a deep groove ventral of the eyes. Head with deep clypeal suture and two pairs of long setae as characteristic for the genus; frons shining and without punctures. Shape of the antennomeres see Fig. 3. Pronotum subquadrate, widest in the middle, anterior and posterior angles completely rounded, anterior and posterior margin finely bordered, lateral margins with broader border, anterior margin not excavated; surface slightly convex, with fine and sparse punctation, punctures only weakly impressed, surface between punctation shining and only slightly shagreened; propleura shining, without punctation. Elytra with nine punctural rows in distinct striae, these punctures small and elongate, only slightly broader than striae, without setae; intervals convex, shining and without punctures nor tubercles, interval 7 at base near shoulders with a longer seta. Ventrites shining, ventrites 1–4 in the middle with a pair of longer setae, last ventrite in both sexes unbordered. Femora and tibiae in both sexes without teeth or other modifications. In males protarsi only slightly dilatated, without other external differences. Aedeagus see Fig. 11.

##### Etymology.

Named in honour of Jean Paul Ardoin (1918–1978), former pharmacist in Arcachon (France), author of the Malagasy genera of Laenini and specialist of other tenebrionids from Africa and Madagascar.

#### Hovadelium bremeri sp. n.

http://zoobank.org/1F59CF9F-4AF5-4510-9915-F1BB12C6CFAA

http://species-id.net/wiki/Hovadelium_bremeri

Figs 4 , 12

##### Type specimens.

Holotype male: E Madagascar, 30 km ESE Betroka, Vohitrosa Forest, 1400–1500 m, 17.–18.XII.1998, leg. J. Janák, SMNS. – Paratypes: Same data as holotype, 7 ex. SMNS, 2 ex. ZSM.

##### Diagnosis.

*Hovadelium bremeri* sp. n. and *Hovadelium ardoini* sp. n. are similar, both share the general body shape, the lacking of a groove ventral of the eyes, the shining surface of pronotum and elytra, the elytral interval 7 at base near shoulders with a longer seta, and the lacking tubercles on the elytral intervals. In *Hovadelium bremeri* sp. n., the body length is somewhat shorter in the average, the pronotum is narrower towards base, the pronotal punctation is larger and denser, the anterior pronotal margin is unbordered in the middle, the punctures of the elytral rows are larger, and the apicale of the aedeagus is shorter. See also under *Hovadelium ardoini* sp. n. and key below.

##### Description.

Body length 2.8–3.5 mm, unicoloured dark brown. Eyes flat, not prominent; without a deep groove ventral of the eyes. Head with deep clypeal suture and two pairs of long setae as characteristic for the genus; frons shining and without punctures. Shape of the antennomeres see Fig. 4. Pronotum subquadrate, widest in the middle, anterior and posterior angles completely rounded, anterior margin unbordered in the middle, posterior margin finely bordered, lateral margins with broader border, anterior margin not excavated; surface slightly convex, with irregular larger, but not confluent punctation, punctures only weakly impressed, surface between punctation shining; propleura shining, without punctation. Elytra with nine punctural rows in weak striae, these punctures large and broader than striae, without setae; intervals convex, shining and without punctures nor tubercles, interval 7 at base near shoulders with a longer seta. Ventrites shining, ventrites 1–4 in the middle with a pair of longer setae, last ventrite in both sexes unbordered. Femora and tibiae in both sexes without teeth or other modifications. In males protarsi only slightly dilatated, without other external differences. Aedeagus see Fig. 12.

##### Etymology.

Named in honour of Prof. Dr. H. J. Bremer (Osnabrück, Germany), who provided me with most of the newly collected specimens, and allowed to keep the larger part in SMNS.

#### Hovadelium discoidale

Ardoin, 1961

http://species-id.net/wiki/Hovadelium_discoidale

Fig. 5

##### Reexamined type-material.

“Madagascar” (without detailed data), collection Oberthuer, male holotype MNHN.

##### Remarks.

The type specimen has a quite unique character, namely the head with a deep groove ventral of the eyes (Fig. 5). Ardoin (1961) assumed that this might be a sexualdimorph character of males within the genus. However, in all the plenty herein presented males and females of other species of *Hovadelium*, such a groove is not present. Thus, this structure (of unknown biological function) is considered as not generic but just as specific for *Hovadelium discoidale*. Unfortunately, an exact type locality is unknown.

##### Diagnostic characters.

Body length 4.2 mm (not 5.0 mm as given by Ardoin 1961). Head with a deep groove ventral of the eyes (Fig. 5). Pronotum subquadrate, widest somewhat behind the middle, with marked anterior and rounded posterior angles, surface slightly convex, with fine and sparse punctation, between punctation slightly shagreened. Elytra with punctural rows in striae, intervals convex, shining and without tubercles, interval 7 at base near shoulders with a longer seta. Aedeagus not examined herein (because of the fragility of the type).

#### Hovadelium elongatum

Ardoin, 1976

http://species-id.net/wiki/Hovadelium_elongatum

Figs 6 , 13

##### Reexamined type-material.

None, not found in MNHN.

##### Type locality.

SE Madagascar, Plateau Andohahelo, SE Trafonaomby, 1770–1950 m.

##### New material.

SE Madagascar, 3 km NW Fort Dauphin, Pic St. Louis, 150–250 m, 19.II.2004, leg. P. Bulirsch, 6 ex. CRFL, 2 ex. SMNS. – E Madagascar, 38 km ESE Betroka, Kalambatrita Forest, 3 km SSE Ambaro, 1400 m, 29.XII.1998, leg. J. Janák, 2 ex. SMNS.

##### Remarks.

I hope not to fail in assigning the newly collected specimens to this species, described upon a single female. Distinct differences between the description and the new specimens do not exist, and the larger part of the new material originates from the surroundings of the type locality nearby Fort Dauphin.

##### Diagnostic characters.

Body length 4.0–6.0 mm (holotype 5.0 mm). Pronotum cordiform, anterior margin regularly excavated, widest before the middle, with rounded anterior and posterior angles, surface slightly convex, with fine and sparse punctation, between punctation distinctly shagreened. Elytra with punctural rows in striae, intervals convex, slightly shagreened and somewhat uneven (“petit granules peu saillants” in description), interval 7 at base near shoulders with a longer seta. Aedeagus see Fig. 13.

**Figures 6–9.**
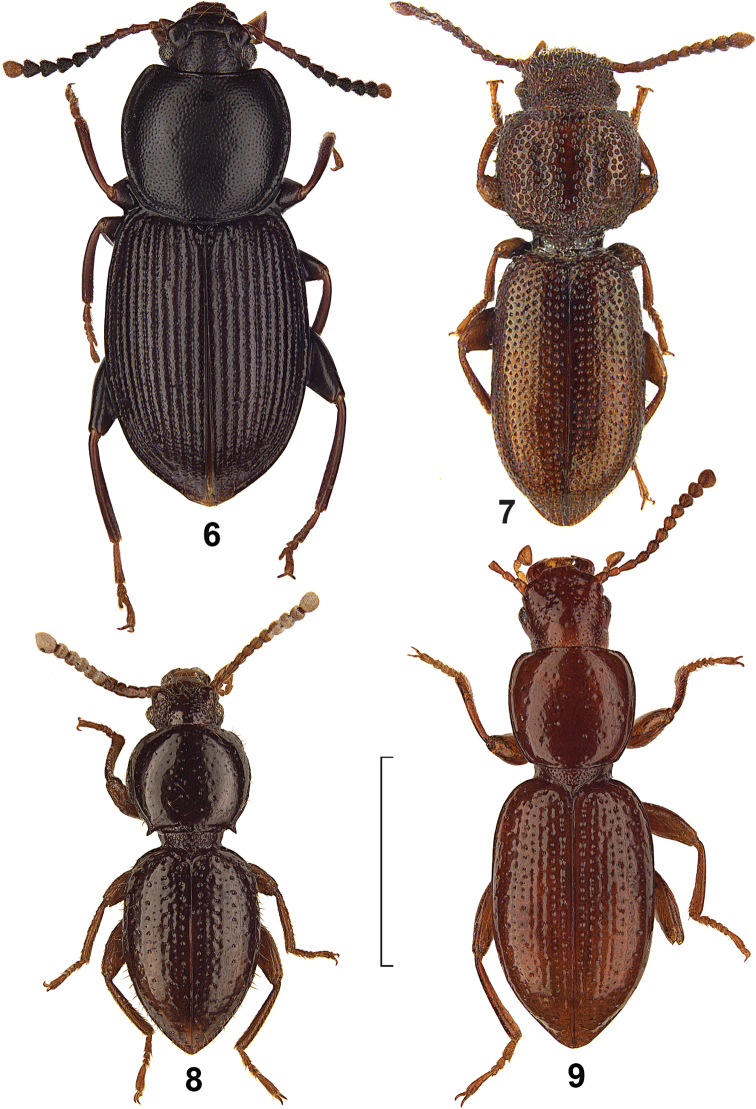
Dorsal view of *Hovadelium* and *Mimolaena* species. **6** *Hovadelium elongatum*, non-type SMNS **7** *Mimolaena clarissae*, holotype MZUF **8** *Mimolaena janaki* sp. n., holotype SMNS. **9** *Mimolaena pauliani*, holotype MNHN. – Scale line 2 mm.

**Figures 10–14.**
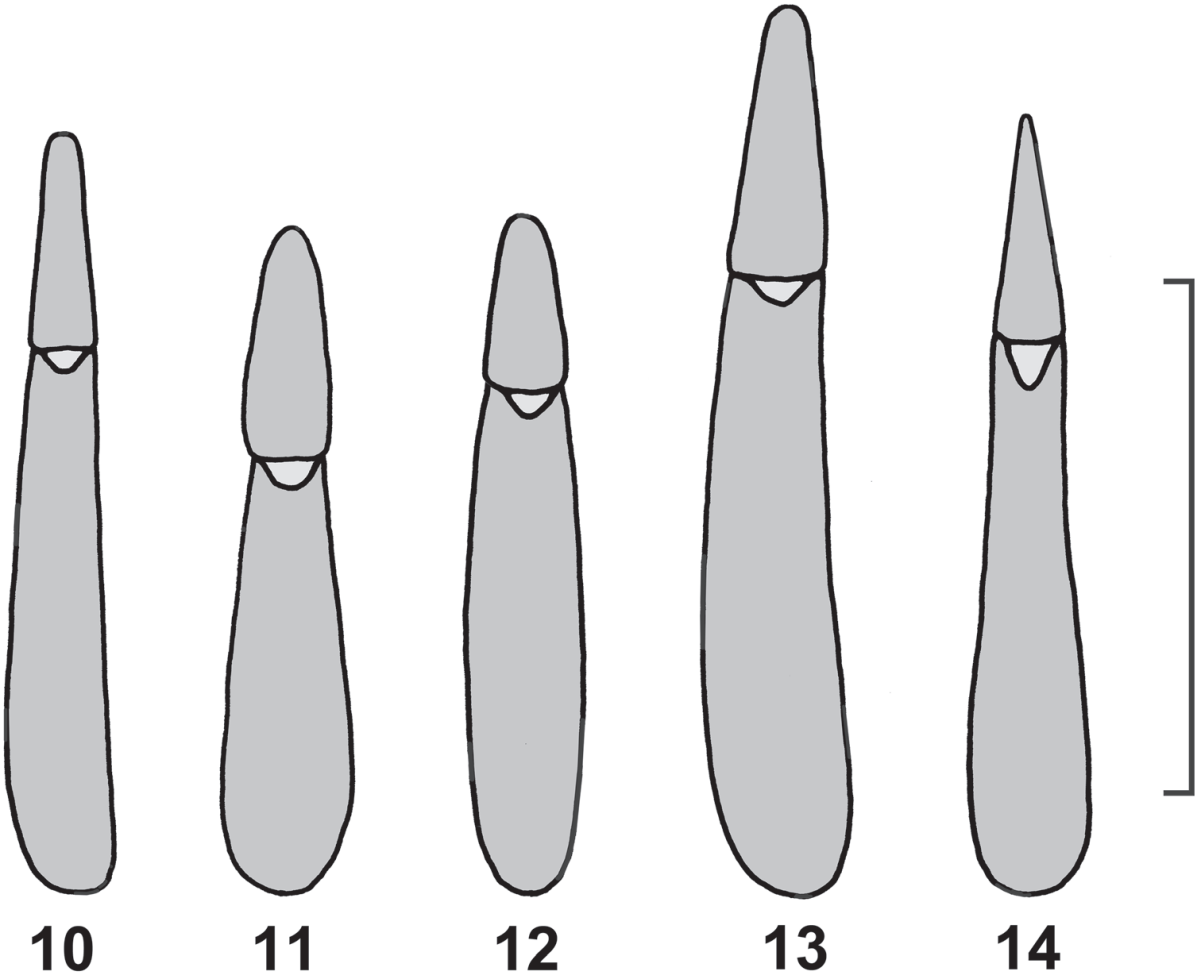
Aedeagus of *Hovadelium* and *Mimolaena* species. **10** *Hovadelium aeneum*, non-type SMNS **11** *Hovadelium ardoini* sp. n., holotype SMNS **12** *Hovadelium bremeri* sp. n., holotype SMNS **13** *Hovadelium elongatum*, non-type SMNS **14** *Mimolaena janaki* sp. n., holotype SMNS. – Scale line 0.5 mm.

**Figure 15.**
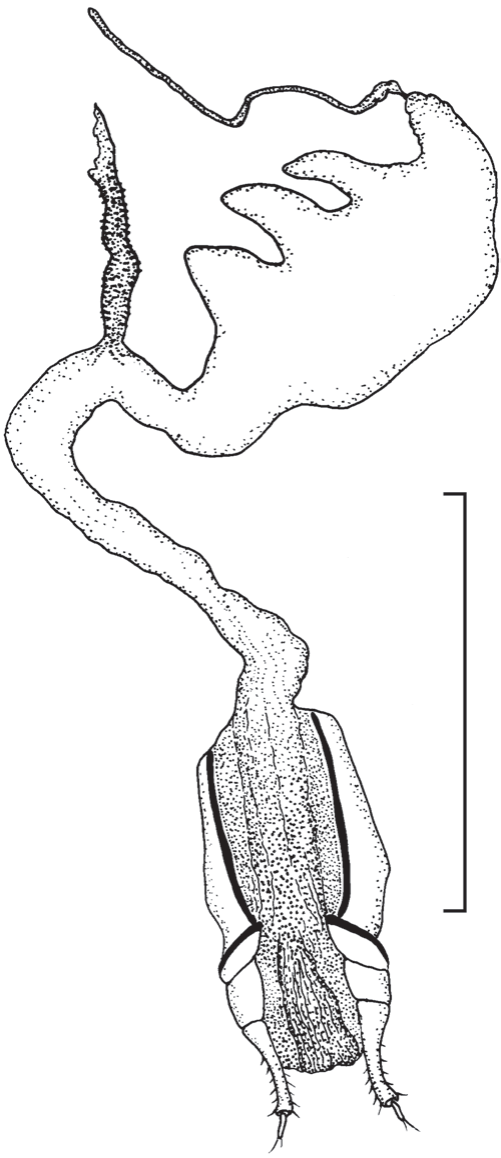
Ovipositor and female genital tract of *Hovadelium ardoini* sp. n. (drawing by Eric Matthews). – Scale line 1 mm.

### The genus *Mimolaena*

#### Mimolaena clarissae

Ferrer, 1998

http://species-id.net/wiki/Mimolaena_clarissae

Fig. 7

##### Reexamined type-material.

E Madagascar, Ambila Lemaitso (labelled as Ambila La Maintso), V.1990, leg. C. Raharimina, female holotype MZUF.

##### Diagnostic characters.

Body length 4.4 mm. Pronotum subquadrate, with slightly prominent anterior and with rounded posterior angles, surface slightly convex and with rough and partly confluent punctures. Elytra with rough irregular punctation without any separation in rows and intervals. Aedeagus unknown, only ♀ holotype known.

#### Mimolaena janaki sp. n.

http://zoobank.org/CB2004A1-7F5A-423C-99C7-AF4BAC9E1EE3

http://species-id.net/wiki/Mimolaena_janaki

Figs 8 , 14

##### Type specimens.

Holotype male: E Madagascar, Ranomafana NP, Vohiparara, 1100–1200 m, 21.–24.I.1993, leg. J. Janák, SMNS. – Paratypes: Same data as holotype, 3 ex. SMNS, 2 ex. ZSM. – E Madagascar, Massiv Ambondrombe, 1600–1700 m, 17.III.1996, leg. J. Janák & P. Moravec, 1 ♀ SMNS. – E Madagascar, Massiv Ambondrombe, 1500–1600 m, 15.–18.III.1996, leg. J. Janák & P. Moravec, 1 ♀ ZSM. – E Madagascar, Massiv Ambondrombe, 1300–1400 m, 14.III.1996, leg. J. Janák & P. Moravec, 1 ♀ SMNS.

##### Diagnosis.

To be recognized by the shape of the pronotum with spine-like posterior angles, by scattered and fine punctation of the pronotum, by only six elytral rows of punctures extinguished in the posterior and external part of elytra, and by the shape of the aedeagus. The two other known species of *Mimolaena* possess nearly rounded posterior angles of the pronotum, and the punctation on the pronotum is either fine and the elytra bear punctural rows (*Mimolaena pauliani* Ardoin, 1961), or the punctation on the pronotum is rough and dense and the elytra bear an dense irregular punctation not separated in rows and intervals (*Mimolaena clarissae* Ferrer, 1998). See also key below.

##### Description.

Body length 3.5–4.7 mm, unicoloured dark brown. Eyes (Fig. 8) not reduced, slightly prominent. Shape of the antennomeres see Fig. 8. Shape of pronotum see Fig. 8, disc with a few scattered punctures, most punctures bearing a longer erect seta; surface without any impressions, surface shining, lateral margins bordered, basal margin bordered and not bent downwards, posterior angles prominent spine-like, propleura unpunctured. Elytra (Fig. 8) with only six punctural rows without striae, these rows extinguishing in posterior and external part of elytra, punctures of rows of similar size as pronotal punctures, punctures of the elytral rows without setae, a few additional punctures apart from the rows laterally and distally on the elytra bear a longer erect seta, intervals shining without any punctures and setation, intervals flat. Ventrites shining, in males with fine punctation and short setation, in females nearly unpunctured and without setation, last ventrite in both sexes unbordered. Femora and tibiae in both sexes without teeth or other modifications. Aedeagus see Fig. 14.

##### Etymology.

Named in honor of J. Janák, one of the collectors of the type series and of other Malagasy Laenini.

#### Mimolaena pauliani

Ardoin, 1961

http://species-id.net/wiki/Mimolaena_pauliani

Fig. 9

##### Reexamined type-material.

C Madagascar, Plateau Soaindrana, Andringitra-Ambalavao, 2090 m, 16.I.1958, leg. R. Paulian, male holotype and 1 male paratype MNHN.

##### Diagnostic characters.

Body length 4.4–4.8 mm. Pronotum subquadrate, with rounded anterior and posterior angles, surface flat and with sparse and fine punctation. Elytra with distinct punctural rows without striae, size of the punctures diminishing laterally and apically. Aedeagus not examined herein (because of the fragility of the type). It is said (but not figured) in the description, that the apicale is short and acute at the tip, and the basale is long and bent.

### Identification key of Laenini from Madagascar

**Table:** 

1	Base of elytra excavated for pronotum, humeral angle protruding; head between eyes and clypeus each with a pair of long tactile setae; elytral interval 7 at base near shoulders with a long tactile seta (genus *Hovadelium*)	2
–	Base of elytra not excavated, humeral angle rounded; head with irregular short setation, without pairs of long setae; elytral interval 7 at base near shoulders without a long tactile seta (genus *Mimolaena*)	6
2	Head with a deep groove ventral of the eyes – Fig. 5	*Hovadelium discoidale*
–	Head “normal”, without groove	3
3	Pronotum cordiform, surface of head and pronotum distinctly mat and shagreened – Fig. 6	*Hovadelium elongatum*
–	Pronotum subquadrate, surface of head and pronotum shining or at most weakly shagreened	4
4	Pronotum widest before the middle, elytral intervals with an irregular row of tubercles – Fig. 2	*Hovadelium aeneum*
–	Pronotum widest in the middle, elytral intervals without tubercles	5
5	Lateral margin of pronotum regularly rounded towards anterior and posterior angles, anterior margin of pronotum completely bordered, pronotal punctation fine, punctures of elytral rows small, elongate and not distinctly broader than distinct striae – Fig. 3	*Hovadelium ardoini* sp. n.
–	Lateral margin of pronotum narrower towards base, anterior margin of pronotum unbordered in the middle, pronotal punctation larger and denser, punctures of elytral intervals larger and broader than weak striae – Fig. 4	*Hovadelium bremeri* sp. n.
6	Posterior angles of pronotum prominent spine-like – Fig. 8	*Mimolaena janaki* sp. n.
–	Posterior angles of pronotum rounded	7
7	Pronotum with fine and separate punctation, elytra with punctural rows – Fig. 9	*Mimolaena pauliani*
–	Pronotum with rough and partly confluent punctation, elytra with rough irregular punctation without any separation in rows and intervals – Fig. 7	*Mimolaena clarissae*

## Supplementary Material

XML Treatment for Hovadelium aeneum

XML Treatment for Hovadelium ardoini

XML Treatment for Hovadelium bremeri

XML Treatment for Hovadelium discoidale

XML Treatment for Hovadelium elongatum

XML Treatment for Mimolaena clarissae

XML Treatment for Mimolaena janaki

XML Treatment for Mimolaena pauliani
